# Is It Time to Explore the Health Policy Process Within Governance and Health Systems Frameworks? Comment on "Modelling the Health Policy Process: One Size Fits All or Horses for Courses?"

**DOI:** 10.34172/ijhpm.2023.8047

**Published:** 2023-06-18

**Authors:** Stephen Peckham

**Affiliations:** Centre for Health Services Studies, University of Kent, Canterbury, UK

**Keywords:** Health Policy Analysis, Policy Theory, Policy Models

## Abstract

There is clearly a need to improve the use of more robust policy theory on health policy analysis. Powell and Mannion in an editorial on the relationship between health policy analysis and the wider field of public policy theory note, as others have done before, the limited application of policy theory in health policy analysis. However, they also highlight that within the health policy analysis arena new models have emerged which have wider use within policy analysis such as the health policy triangle. While Powell and Mannion suggest that health policy analysis can take one of two paths I argue that we should be developing more integrated frameworks of health policy processes, governance and systems which would involve the use of robust public policy theories and models.

## Introduction

 In their editorial Powell and Mannion^1^ suggest that the *“… literature on the health policy process is semi-detached from the wider policy process literature*” (p. 1).They discuss a number of approaches adopted in health policy analysis and compare these to the wider policy process literature in an attempt to determine whether this disconnect really exists. They are not the first analysts to consider how policy models and theories are used in practice. John^2^ has highlighted the abundance of approaches to explaining and understanding policy-making and noted that there is little agreement about what a model of the policy process should look like. For Powell and Mannion^1^ the key question is whether analysis of the health policy process constitutes a sufficiently distinct and self-contained area of analysis. They suggest that *“There seem to be two very different future research directions: focusing on ‘home grown’ models, or taking greater account of the wider policy process literature”* (p. 4) asking *“Does ‘one size fit all’ or is it ‘horses for courses’?”* (p. 4). Their analysis is, however, seemingly based on a narrow range of examples as there is a specific focus on “models” AND ‘policy’ and ‘health’ (p. 2). In fact the wider health policy literature, particularly analyses of health policy in lower- and middle-income countries is characterised by a very rich policy and political analysis literature,^3^ while other analysts have drawn on political and organisational, governance and systems theories.^4-8^ There are also significant gaps in their discussion in terms of the influence of theories such as structured interests (Alford), Street Level Bureaucrats (Lipsky), the sociology of organisations (Perrow) and professional power (Freidson) that have been used in health policy analysis.

## Defining the Scope of Health Policy Analysis

 Rather than unpick their arguments in detail I want to suggest that there is another way of considering this and argue that neither a *‘one size fit all’ *nor a *‘horses for courses’ *approach is satisfactory. Most analyses of policy rightly draw on multiple policy lenses.^9^ Simply referring to either applied health policy process models or stronger use of public policy theory, a more useful approach would be to combine such approaches within governance and systems analyses that provide useful frames for employing robust public policy theory models that are situated within a health context. In doing so I concur with Powell and Mannion and the findings of previous reviews highlighted in the editorial as well as others.

 The first question to address is whether health policy is intrinsically different from other fields of policy and thus has different analytical needs. While it is possible to identify specific elements about health in terms of medical care, different actors and the roles of different actors; similar arguments may also be made about other complex areas of public policy such as education or planning. However, Walt et al^10^ argue that there are specific characteristics which affect the health policy environment and differentiate it from other policy sectors. They cite a number of key distinctive elements of health policy including the political environment, multiple state roles and information asymmetry plus the fact that context varies between low- and higher-income countries and that *“… the policy environment is increasingly populated by complex cross-border, inter-organizational and network relationships, with policies influenced by global decisions as well as by domestic actions”* (p. 309). But does this “special context” demand a separate approach to policy analysis? Weible and Cairney^11^ describe the policy process as being *“… inherently messy and marked by a sticky resistance to change. It is also diverse across contexts and constantly evolving over time”* (p. 194). I am sure there are many analysts who would argue that their special area of policy is subject to complex cross-border, inter-organizational and network relationships, with policies influenced by global decisions as well as by domestic actions – environmental policies, trade and finance spring to mind. Despite this I would argue that context is important — but not in relation to whether health policy analysis is different from applying theory in other policy contexts — therefore making the use of policy theory different, but rather that policy analysis devoid of context is problematic. This point was central to Walt and Gilson’s^12^ policy triangle which highlights the interactions of key pillars, actors, the use of power and the institutional frameworks of health systems. It is unfortunate that while in their editorial Powell and Mannion refer to health policy generally and distinguish this from analysis of “…* particular sub areas or policy domains such as public health, health promotion, tobacco control, obesity, social determinants of health (SDH), geographical areas such as low- and middle-income countries (LMICs) ‘Developing Countries’ or sub-Saharan Africa.”*^1^ However, for much of the rest of the paper they refer to Healthcare policy — itself a sub-area or sub-policy domain within a broader view of health policy.

 Since the development of policy theory in the 1950s there have been tensions between more theory-based research and applied policy research — an area I would argue that health policy falls into. As stated earlier, I support Powell and Mannion’s conclusion that health policy analysts should draw more on general policy theory. Such a conclusion has also been drawn by others for low-, middle-, and high-income countries in reviews by Walt et al,^10^ Gilson and Raphaely,^13^ and Weible and Cairney.^11^ Rather than focus on the need for cross-fertilisation between policy science driven theories and the theoretical development of more applied policy analysis. I think this observation is important as application of policy theory in applied circumstances helps develop stronger and more robust policy theory.

## Extending the Theoretical Armoury for Health Policy Analysis

 Weible and Cairney^11^ advocate a new agenda for policy theory and analysis which *“… requires a reciprocal exchange of information where theories are used to help shape and inform how policy actors understand and act in policy processes, and, in turn, these same people shape and inform the substance, validity and value of theory-based knowledge”* (p. 194). Rather than see the alternative directions identified by Powell and Mannion^1^ I would support an approach that ensures an engagement of health policy analysts with not just wider public policy theory but extend this to wider theoretical analyses such as of the State, governance and complex systems. In this sense I agree the need for health policy analysts to engage in applying, testing and contributing to the development of more robust policy theory and models but health policy analysts need to cast their vision beyond even public policy models to provide clearer analyses and provide richer understandings of policy processes. Weible and Schlager^14^ argue that policy analysis should move beyond case studies of single policies to analyse and interpret broader policy areas. Compton and ‘t Hart^15^ have highlighted the importance of examining the temporal aspects of policy change. These are as many challenges to health policy analysts and to those scholars developing policy theory. Otherwise, we are at risk of developing health systems research yet again detached from the theories that seek to understand the nature of the policy process.

 Powell and Mannion limit the discussion to an analysis of specific policy models and theories used in health policy analysis, but this is a reflection of the limited use of policy models in studies examining the policy process. While their review highlights the findings of previous attempts to examine the use of policy theory bemoaning the limited application of policy theory models and methodological limitations it does not engage with a much broader and relevant literature. While not necessarily sitting in the health policy analysis canon other studies of policy change have drawn on substantive theoretical approaches that are clearly relevant to understanding the policy process by situating policy theories in broader contextual frameworks. For example, a review of theory use could have considered contributions of analysts such as Gestel et al^4^ and Tuohy^5^ who draw on institutional theory and some analysts of health policy have explored the application of wider frameworks such as Wu and colleagues’^16^ policy capacity framework and the increasing application of Health Systems theory to contextualise and frame the use of policy theories within the health field or provide explanations of policy change.^6,7^ The framework was reconfigured to understand modes of governance as a nested set of capacities and, therefore, favoured a comprehensive approach to policy analysis in various public sectors but as yet, as demonstrated perhaps by their absence in Powell and Mannion’s editorial, not been widely used in health policy analysis.^15^

 The more policy analysts working in applied fields of analysis such as health policy draw on these broader systems and governance frameworks and engage with policy theory research the richer the understandings of policy process will be. Schlager^17^ noted the shortcomings of analysis that draws on theories and models as discrete approaches pointing out that the study of policy analysis has been characterised by “*mountain islands of theoretical structure, intermingled with and occasionally attached together by foothills of shared methods and concepts, and empirical work, all of which is surrounded by oceans of descriptive work not attached to any mountain of theory” *(p. 14). Health policy could be portrayed as the “*foothills of shared methods and concepts, and empirical work” *detached from more theory-based policy research. She highlighted the value of drawing on different but complimentary analytical lenses to examine unfolding policy. There is then the potential for health policy analysts to employ a range of robust policy theory within system and governance framework that seek to contextualise health policy. In fact the use of systems and governance frameworks can be seen across the broad range of policy analysis fields.

## Shifting the Health Policy Analysis

 Increasingly, and importantly, policy analysts are seeking explanations not just of discrete elements of policy but wanting to understand why policy leads to certain outcomes requiring an understanding of how policies are designed, selected, implemented and what their impact is. An approach that focuses on “home grown” models may not always be helpful in terms of developing generalisable theories or frameworks. As Walt and Gilson have argued analysts need to link together design and implementation given the clear recognition of the non-linear nature of the policy process.^12^ Health systems perspectives or longer temporal views of policy change ought to explicitly draw the analyst away from the use of single frameworks or models such as Multiple Streams or Advocacy Coalitions to developed more nuanced analysis that is by definition broader in scope. To understand the development and implementation of health policy, analysis must be both context dependent and theoretically derived. Policy analysis needs to be rooted both in theories about institutional and health system governance.^6^ There has been a recent resurfacing of interest in policy success and failure and starting to understand the complexities of the reasons for policy outcomes. A rigid reliance on particular models of process and simple empirical analysis of health discrete policies may have little to offer policy-makers in terms of how to improve policy without engaging in new perspectives such as policy capacity and health systems analysis. I am not convinced that the call in the paper for better use of policy theory in applied analysis does suggest *“… future directions for research on this topic.”* I would argue that a key area for more applied and theoretical development is on the intersection of policy theory and areas such as policy capacity frameworks and systems theory. Such approaches, seen in particular in application in low- and middle-income countriescan provide new and richer ways of understanding policy processes.

## Ethical issues

 Not applicable.

## Competing interests

 Author declares that he has no competing interests.
